# Mental health treatment and its impact on survival outcomes in patients with comorbid mental health and cardiovascular diseases: a retrospective cohort study

**DOI:** 10.1186/s12888-025-07035-4

**Published:** 2025-07-01

**Authors:** Tirsit Ketsela Zeleke, Fasil Bayafers Tamene, Gebremariam Wulie Geremew, Tekletsadik Tekleslassie Alemayehu, Minichil Chanie Worku, Samuel Agegnew Wondm, Aschalew Mulatu Tefera, Ayelign Eshete Fitgu, Wubetu Yihunie Belay, Abraham Teym, Rahel Belete Abebe, Bantayehu Addis Tegegne

**Affiliations:** 1 Department of Pharmacy, College of Health Sciences, Debre Markos University, Debre Markos, Ethiopia; 2 Department of Clinical Pharmacy, School of Pharmacy, College of Medicine and Health Sciences, University of Gondar, Gondar, Ethiopia; 3 Department of Social and administrative Pharmacy, School of Pharmacy, College of Medicine and Health Sciences, University of Gondar, Gondar, Ethiopia; 4 Department of Pharmaceutical Chemistry, College of Medicine and Health Sciences, University of Gondar, Gondar, Ethiopia; 5 Department of Pharmacognocy, College of Medicine and Health Sciences, University of Gondar, Gondar, Ethiopia; 6 Department of Environmental Health, College of Health Sciences, Debre Markos University, Debre Markos, Ethiopia

**Keywords:** Mental health treatment, Cardiovascular diseases, Hospital readmissions, Emergency department visits, Comorbid mental health and cardiovascular diseases, Ethiopia

## Abstract

**Background:**

Mental illness and cardiovascular diseases frequently co-occur and are among the leading causes of global morbidity and mortality. Their comorbidity is associated with poorer health outcomes, including higher mortality, hospital readmissions, and increased healthcare utilization. Although mental health treatment has been shown to improve clinical outcomes, its impact on patient survival outcomes remains underexplored. This study aims to evaluate the effects of mental health treatment on hospital readmissions, emergency department visits, and overall survival time in patients with comorbid mental health and cardiovascular conditions.

**Methods:**

A multi-center retrospective cohort study was carried out among adult comorbid mental health and cardiovascular diseases patients in Ethiopia. Data entry was performed using EpiData Manager, and the dataset was subsequently exported to SPSS version 26 for analysis. A Cox proportional hazards regression model was applied to identify factors influencing the time to hospital readmission and emergency department visits. Adjusted hazard ratios with corresponding 95% confidence intervals were reported, and statistical significance was determined at a *P*-value threshold of < 0.05. Kaplan-Meier survival curves were used to illustrate differences in time to hospital readmission and emergency department visits between treated and untreated patients.

**Results:**

Depression was the most prevalent mental health condition, affecting 47.3% of participants, while hypertension was the most common cardiovascular illness in 37.3% of participants. The rate of mental health treatment in this study is 35.7%. Determinates of hospital readmission included mental health treatment AHR 3.44 (95% CI: 2.11–5.62) and the presence of comorbid conditions AHR of 1.53 (95% CI: 1.03–2.28). Additionally, emergency department visits were significantly associated with mental health treatment AHR of 2.11 (95% CI: 1.09–4.08). Kaplan-Meier survival curves indicated that patients receiving mental health treatment experienced longer times to readmission and emergency department visits compared to untreated patients.

**Conclusions:**

Mental health treatment is associated with improved survival outcomes and reduced hospital readmissions and emergency department visits, in patients with comorbid mental health and cardiovascular diseases. These findings indicate the importance of integrating mental health care into the management of patients with complex medical conditions to improve long-term outcomes and reduce the burden on healthcare systems. Moreover, it is important to pay attention to patients with comorbid diseases.

**Clinical trial number:**

Not applicable.

**Supplementary Information:**

The online version contains supplementary material available at 10.1186/s12888-025-07035-4.

## Introduction

Mental health disorders and cardiovascular diseases (CVDs) are two of the most prevalent health conditions worldwide, contributing significantly to overall morbidity and mortality [1, 2]. The interrelationship between mental health and cardiovascular health has gained increasing recognition, with a well-documented bidirectional connection between the two [3, 4]. The term comorbid mental health and cardiovascular diseases refers to the co-occurrence of both mental health disorders and cardiovascular diseases in a single patient [5]. These conditions often interact in complex ways: mental health disorders can influence the development, progression, and outcomes of cardiovascular diseases, and vice versa [6, 7, 8]. Focusing on individuals with coexisting mental and cardiovascular conditions is essential, as these patients tend to experience worse clinical outcomes, increased healthcare utilization, and greater barriers to care compared to those with either condition alone [7, 9]. The dual burden further complicates management, particularly in resource-limited settings where healthcare systems are often fragmented and ill-equipped to manage multimorbidity effectively.

Patients with mental health disorders including depression, anxiety, bipolar disorder, and schizophrenia, are at an elevated risk of developing cardiovascular conditions such as hypertension, coronary artery disease, heart failure, atrial fibrillation, and stroke [10, 11, 12]. These patients are more likely to engage in unhealthy behaviors such as smoking, physical inactivity, and poor dietary habits, which increase cardiovascular risk. Furthermore, the chronic stress associated with mental illness can exacerbate cardiovascular conditions through physiological mechanisms such as inflammation and autonomic dysregulation [13, 14]. Conversely, individuals with cardiovascular diseases often experience psychological distress due to the chronic nature of their condition, functional limitations, and the perceived threat to life [15]. Cardiovascular diseases are responsible for approximately 17.9 million deaths annually, accounting for 32% of all global deaths, 85% of which result from heart attacks and strokes [16]. Simultaneously, mental health disorders, particularly depression and anxiety, are leading contributors to disability-adjusted life years worldwide [17]. This dual burden is particularly pronounced in low- and middle-income countries such as Ethiopia [18, 19], where healthcare systems face challenges in providing adequate care for both mental and cardiovascular conditions. Globally, only a small percentage of individuals with mental illness receive evidence-based treatment, with the majority of untreated cases found in low-income countries. In Ethiopia, fewer than 10% of people with mental disorders receive any form of treatment, and even fewer access care integrated with physical health services [20].

Ethiopia faces additional challenges that intensify the burden of comorbid mental and cardiovascular diseases. Mental health services are scarce, with specialized psychiatric care largely concentrated in urban areas, leaving rural populations with minimal access [21]. Cultural stigma surrounding mental illness further hinders help-seeking behavior, contributes to under diagnosis, and negatively affects treatment adherence [20]. Historically, the healthcare system has prioritized acute infectious diseases, with limited attention given to non-communicable diseases and even less to mental health integration within chronic disease management [18]. These systemic, cultural, and infrastructural barriers result in fragmented care, high unmet treatment needs, and elevated morbidity and mortality among patients with coexisting mental and cardiovascular conditions. Investigating the role of mental health treatment in this setting is thus critical for informing interventions aimed at improving patient outcomes in Ethiopia and similar low-resource environments. While mental health treatment is known to enhance psychological well-being, emerging evidence also highlights its impact on physical health outcomes. For instance, appropriate treatment of CVD has been shown to significantly improve survival in patients with comorbid psychiatric conditions [22]. Yet, individuals with mental illness often receive suboptimal cardiovascular care, including underuse of evidence-based medications like statins and beta-blockers, as well as limited access to cardiac interventions, factors that contribute to excess mortality [23]. However, access to guideline-directed cardiovascular therapies combined with mental health support has been associated with better prognoses, reduced hospitalizations, and improved survival among patients with comorbid conditions [24]. These findings emphasize the importance of integrated treatment strategies addressing both mental and cardiovascular health.

Mental health interventions including pharmacotherapy and psychotherapy have been shown to reduce both psychological well-being and physical health outcomes [25]. Pharmacotherapy for mental health disorders has been associated with reduced depressive and psychotic symptoms and improved adherence to cardiovascular medications [22, 26]. In high-income countries, integrated care models targeting both mental and physical health have led to reductions in hospital readmissions, improved blood pressure control, and lower mortality rates [27].

Despite these established benefits, there is a significant gap by evaluating the impact of mental health treatment on survival outcomes, hospital readmissions, and emergency department visits in patients with comorbid mental health and cardiovascular conditions in Ethiopia. Given the high burden of both mental and cardiovascular diseases and the limited availability of mental health services, there is an urgent need to examine whether mental health treatment improves survival and reduces hospital utilization in this context. This study aims to fill that gap by evaluating the impact of mental health treatment on survival outcomes, hospital readmissions and emergency department visits in patients with comorbid mental health and cardiovascular diseases in Ethiopia. Specifically, the primary objective is to assess whether receiving mental health treatment is associated with improved survival outcomes. We hypothesize that patients receiving mental health treatment will experience a fewer hospital readmissions, fewer emergency department visits, and longer survival times compared to untreated patients. This study will contribute to the limited literature on the relationship between mental health treatment and patient health outcomes, particularly in the context of Ethiopia where the dual burden of diseases poses challenges. The findings could inform clinical practices, influence health policy, and provide a basis for integrated care models aimed at improving the health and well-being of individuals with comorbid mental and cardiovascular diseases in Ethiopia and similar settings.

## Methods and materials

### Study setting

This study was conducted at four major healthcare facilities in Northwest Ethiopia including Debre Markos Comprehensive Specialized Hospital, Tibebe Gihon Comprehensive Specialized Hospital, University of Gondar Comprehensive Specialized Hospital, and Felege Hiwot Comprehensive Specialized Hospital.

### Study period and design

The study was conducted, from January 1, 2023, to May 31, 2023. A retrospective cohort design was employed to assess existing medical records, using a one year dataset.

### Source and study population

The study population consisted of patients diagnosed with comorbid mental health and cardiovascular diseases who received care at the participating hospitals. Patients were identified through hospital admission and discharge records, outpatient clinic logs, and electronic health records.

### Eligibility criteria

The inclusion criteria for participants were:

- A confirmed diagnosis of comorbid mental health and cardiovascular disease in medical records.
- Age 18 years or older at the time of diagnosis.
- Available medical records for the duration of the study period.

The exclusion criteria for participants were:

- Patients with incomplete medical records,
- Those who had prior cardiovascular surgeries.
- Individuals with terminal illnesses unrelated to comorbid mental health and cardiovascular diseases.

### Study variables

The dependent variables are hospital readmission and emergency department visits. The independent variables included mental health treatment, age, sex, and residence.

### Sample size determination

This study included all patients who met the eligibility criteria during the study period. As the study design was based on medical record review, no a priori sample size or power calculation was performed. Instead, the full population of eligible patients included to maximize statistical power and ensure generalizability.

A total of 319 patients with comorbid mental health and cardiovascular diseases between January 2018 and December 2022 were identified from four healthcare institutions in Northwest Ethiopia. These institutions were selected in simple random approaching method.

### Sampling technique

To ensure the sample was representative of the eligible population across the participating hospitals, a proportional simple random sampling technique was employed. The total number of eligible patients at each hospital during the study period was first identified through a manual review of patient records. Proportional allocation was then used to determine the number of patients to include from each hospital based on its share of the total eligible patient population.

The formula used for proportional allocation was: n_i_ = (N_i_ / N) × n.

Where:

- n_i_ = sample size from hospital *i*.
- N_i_ = number of eligible patients in hospital *i*.
- N = total number of eligible patients across all hospitals.
- n = total sample size (319).

Based on estimated eligible patient numbers from hospital records (*N* = 1,100), the sample was allocated as follows:

- Debre Markos Comprehensive Specialized Hospital: *n*_1_ = (360 / 1100) × 319 ≈ 104 patients.
- University of Gondar Comprehensive Specialized Hospital: *n*_2_ = (300 / 1100) × 319 ≈ 87 patients.
- Felege Hiwot Comprehensive Specialized Hospital: *n*_3_ = (240 / 1100) × 319 ≈ 70 patients.
- Tibebe Gihon Comprehensive Specialized Hospital: *n*_4_ = (200 / 1100) × 319 ≈ 58 patients.

After determining the number of participants per hospital, simple random sampling was applied within each hospital. Eligible patient lists were prepared, and random numbers were generated using a computer-based random number generator to select participants independently.

### Data collection procedure

This study employed a structured questionnaire, developed after an extensive review of relevant literature. The data collection instrument designed to capture sociodemographic characteristics, clinical parameters, and medication-related variables, with all data extracted from patient medical records. Comorbid conditions including diabetes mellitus, hyperlipidemia, hypertension, and other chronic physical conditions were identified based on clinician-documented diagnoses in the medical charts. Comorbidity was considered present if it was recorded in the patient’s medical history, diagnostic summary, or treatment plan during admission or follow-up visits. These conditions were categorized as binary variables (present or absent), and no additional thresholds related to disease severity, duration, or laboratory values were applied due to variability in documentation across sites.

Multiple methodologies were employed to assess the receipt of mental health treatment. Pharmacy refill records were used to determine whether patients actively received prescribed mental health medications during the study period. The duration of these prescriptions was also assessed as a measure of adherence to treatment regimens. Patient charts were systematically examined for indications of mental health treatment, including therapist notes, treatment plans, and mental health evaluations. Specific diagnosis codes associated with mental health conditions were identified to establish a clear connection between diagnosis and treatment. In addition to assessing the receipt of treatment, clinical outcomes related to mental health treatment were analyzed. Indicators such as psychiatric symptoms, changes in diagnoses, and hospitalization rates for mental health crises were assessed. To examine emergency department visits, patient medical records were reviewed throughout the study period. Details such as the reason for each visit, clinical diagnoses, and related mental health assessments were recorded. Visits were categorized based on their connection to comorbid mental health and cardiovascular diseases, mental health crises, or other health complications. For hospital readmissions, a similar review of patient medical records was conducted to track subsequent admissions within a specified follow-up period after discharge. Diagnosis dates were extracted from electronic health records, inpatient and outpatient medical charts, and physician notes. Diagnosis dates were extracted from electronic health records, inpatient and outpatient medical charts, and physician notes. For psychiatric disorders, clinical evaluations, mental health treatment initiation records, and International Classification of Diseases (ICD-10) codes were reviewed, with specific codes. For cardiovascular conditions, diagnostic imaging reports, laboratory results, and physician-confirmed diagnoses, along with corresponding ICD-10 codes, were examined. When exact diagnosis dates were unavailable, the earliest documented evidence of the condition, based on clinical evaluations or treatment initiation was recorded. Given the study’s focus on comorbid mental health and cardiovascular diseases, special attention was given to cases where the timing of psychiatric and cardiovascular diagnoses differed. For patients with pre-existing psychiatric disorders, the timing of the CVD diagnosis was recorded as the key event indicating the onset of a comorbid mental health and cardiovascular diseases. Conversely, for patients with pre-existing CVD, the timing of the psychiatric disorder diagnosis was recorded as the key event. In instances where both conditions were diagnosed simultaneously (e.g., during a single hospital admission), this date was recorded as the timing for both conditions. For patients with multiple episodes of the same condition, such as recurrent depressive episodes or repeated cardiovascular events, the first documented diagnosis within the study period was used.

### Operational definitions

- Comorbid mental health and cardiovascular diseases are health conditions that involve both cardiovascular disorders and psychiatric disorders.
- Mental health treatment refers to interventions aimed at alleviating symptoms and improving the well-being of patients with diagnosed mental health conditions.
- Hospital readmission is defined as any unplanned admission to the hospital. In this study, readmissions included those which are related to comorbid mental health and cardiovascular diseases.
- Emergency department visit is any encounter in the emergency department requiring immediate medical attention. In this study, emergency department visits included those related to comorbid mental health and cardiovascular diseases.
- Event Occurred: refers patients who experienced the outcome of interest during the study period, including those who had a hospital readmission or an emergency department visit.
- Censored: refers patients who did not experience the outcome of interest (hospital readmission or emergency department visit) during the follow-up period. These individuals remained under observation but did not have the event occur before the study’s conclusion or were lost to follow-up.
- Survival time (time to event): This is the duration from the start to the event.

### Data quality assurance

To ensure the integrity and reliability of the data collected in this study, several quality assurance measures were implemented throughout the data collection process. A structured questionnaire was initially developed based on a comprehensive review of relevant literature, to facilitate standardized data capture across all participating institutions. The questionnaire was pre-tested on a small sample of medical records to identify ambiguities, improve clarity, and refine variable definitions prior to full-scale implementation.

Trained research assistants, all of whom were clinical pharmacists, conducted the data extraction. These data collectors underwent rigorous training on the study protocol, ethical considerations, operational definitions, and standard procedures for interpreting medical records. To assess and enhance inter-rater reliability, a pilot exercise was conducted in which 10% of patient charts were independently reviewed by two data collectors. Discrepancies were discussed and resolved through consensus, leading to adjustments in the protocol where necessary. Throughout the data collection period, Periodic supervisory audits were performed. The principal investigator and hospital-based site coordinators randomly reviewed approximately 10% of extracted data to verify accuracy and adherence to protocol. Any inconsistencies were addressed through targeted feedback and retraining sessions with the data collectors. Throughout the study period to ensure compliance with data collection protocols and to address any potential issues promptly. To minimize information bias, diagnoses were confirmed using multiple sources of documentation. Psychiatric disorders were validated by cross-referencing ICD-10 codes with therapist notes, treatment plans, and prescription records. Cardiovascular diagnoses were corroborated using physician-confirmed diagnoses, laboratory and imaging reports, and treatment documentation. In cases where exact diagnosis dates were missing, the earliest documented clinical evidence such as first mention of symptoms or treatment initiation was used as a proxy. To reduce the impact of missing data, records lacking essential variables (e.g., confirmed diagnoses or outcome data) were excluded from analysis. For less critical variables, a complete-case analysis was performed. Given the low frequency of missing data in those variables, imputation methods were not necessary. When feasible, missing details were recovered through triangulation across multiple record sources. To mitigate selection bias, a total population sampling strategy was used. All eligible patients with coexisting psychiatric and cardiovascular conditions who met the inclusion criteria and received care at any of the four participating hospitals were included. Additionally, all medical records and documentation were verified against the entries in the database to confirm accuracy and completeness.

### Data processing and analysis

Data processing and analysis for this study were conducted using statistical software to ensure accurate interpretation of the findings. Following data collection, all questionnaires and medical record entries were reviewed for completeness and consistency. The data were then coded and entered into a secure electronic database to facilitate analysis. Descriptive statistics were generated to summarize the demographic and clinical characteristics of the study population. Categorical variables were described using frequency distributions and percentages, while continuous variables were summarized using means and standard deviations.

To identify factors influencing survival outcomes, Cox proportional hazards regression analysis was performed. The primary outcomes were the time to hospital readmission and the time to the first emergency department visit, both measured in days from the date of discharge or study entry. The follow-up period spanned one year from the date of the first diagnosis or discharge, with censoring applied at the end of the study period or upon loss to follow-up. The assumptions of the Cox proportional hazards model were evaluated using the Schoenfeld residual test. To examine the relationship between baseline variables and patient survival, a two-step approach was employed. Initially, each baseline variable that satisfied the assumptions of the Cox proportional hazards model was analyzed individually using separate Cox regression models. Subsequently, variables with a *P*-value of less than 0.25 in the bivariate analysis were included in the multivariable analysis. However, final inclusion was not based solely on statistical criteria. We also incorporated variables based on their clinical relevance, biological plausibility, and established evidence from prior studies on mental health and cardiovascular outcomes. The Cox regression model was utilized to identify factors associated with the time to hospital readmission and emergency department visit. The results were reported as crude hazard ratios (CHR) and adjusted hazard ratios (AHR) with corresponding 95% confidence intervals, and statistical significance was determined at a *P*-value threshold of < 0.05. Additionally, multicollinearity among the independent variables was assessed using the variance inflation factor to detect and eliminate redundant variables that could bias the estimates. The overall mean VIF was calculated to be 1.21, which falls within the acceptable range of 1 to 5. Survival analysis was further conducted using Kaplan-Meier survival curves to illustrate survival functions, and the log-rank test was applied to compare survival distributions between patients who received mental health treatment and those who did not.

## Result

### Demographic and clinical characteristics of the study participants

A total of 319 patients were included in the study, with males comprising 53.3% of the sample. The average age of the participants was 56 years, with a standard deviation of ± 12.60. Most participants (60.8%) resided in urban areas. Regarding specific psycho cardiovascular diagnosis, depression was the most prevalent mental health illness affecting 47.3% of participants, followed by anxiety (28.0%) and bipolar disorder (14.4%). Among cardiovascular conditions, hypertension was the most common diagnosis (37.3%), followed by coronary artery disease (22.9%) and heart failure (15.0%). The majority of the participants (67.7%) had comorbid diseases. Hyperlipidemia (32.0%) and type 2 diabetes mellitus (23.3%) were the most frequently reported comorbidities, while other notable comorbidities included chronic kidney disease (10.8%) and asthma (8.7%). During the follow-up period, hospital readmission rates showed that 33.9% of patients experienced readmissions during the follow-up period, while 66.1% were censored. Additionally, emergency department visits were recorded for 13.5% of patients, with 86.5% being censored (Table 1).

**Table 1:** Frequency of demographic and clinical characteristics of study participants

	Variables	Frequency (*n*)	Percent (%)
Sex			
	Male	170	53.3
	Female	149	46.7
Age			
	< 56 years	112	35.1
	≥ 56 years	207	64.9
Residence			
	Rural	125	39.2
	Urban	194	60.8
Diagnosis (ICD-10 Codes)			
	Mental health disorders		
	Depression (F32)	151	47.3
	Anxiety (F41)	89	28.0
	Bipolar disorder (F31)	46	14.4
	Schizophrenia (F20)	33	10.3
	Cardiovascular diseases		
	Hypertension (I10)	119	37.3
	Coronary artery disease (I25.1)	73	22.9
	Heart failure (I50)	48	15.0
	Peripheral artery disease (I73.9)	31	9.7
	Atrial fibrillation (I48)	22	7.0
	Stroke (I63)	19	5.9
	Valvular heart disease (I34)	7	2.2
Presence of comorbid conditions			
	No	216	67.7
	Yes	103	32.3
Type of comorbid conditions			
	Type two Diabetes mellitus (E11)	24	23.3
	Type one Diabetes mellitus (E10)	9	8.7
	Chronic kidney disease (N18)	11	10.8
	Chronic obstructive pulmonary disease (J44)	6	5.8
	Osteoarthritis (M15)	3	2.9
	Hyperlipidemia (E78)	33	32.0
	Asthma (J45)	9	8.7
	Hyperthyrodism (E05)	3	2.9
	Epilepsy (G40)	1	1.0
	Gastroesophageal reflux disease (K21)	4	3.9
Hospital readmission			
	Censored	211	66.1
	Occurred	108	33.9
Emergency department visit			
	Censored	276	86.5
	Occurred	43	13.5

### Rate of mental health treatment among comorbid mental health and cardiovascular diseases patients

Regarding mental health treatment, 35.7% of the participants (114 patients) received mental health treatment, while 64.3% did not. Among the patients receiving treatment, 30.7% of patients were prescribed antipsychotics. Haloperidol was the most frequently used antipsychotic, given to 35.6% of patients, followed by chlorpromazine and risperidone which was prescribed in 27.1% and 15.2% of patients. Antidepressants were prescribed to 28.1% of the patients receiving mental health treatment with sertraline being the most common, used by 47.7% of patients. For mood stabilizers, which were prescribed to 23.7% of patients, carbamazepine was the most frequently used (51.3%). In addition to mono therapies, 17.5% of the treated patients received a combination of medications. Of these, the combination of an antipsychotic with a mood stabilizer was the most common, used by 45.0% of patients (Table 2).

**Table 2:** Frequency of mental health treatment related variables among the study participants

	Variable		Frequency (*n*)	Percent (%)
Mental health treatment				
	Not received		205	64.3
	Received		114	35.7
List of medications given for mental health treatment				
	**Antipsychotics**		**35**	**30.7**
		Haloperidol	13	35.6
		Chlorpromazine	9	27.1
		Risperidone	7	15.2
		Clozapine	4	11.9
		Olanzapine	1	6.8
		Quetiapine	1	3.4
	**Antidepressants**		**32**	**28.1**
		Sertraline	16	47.7
		Fluoxetine	11	27.1
		Amitriptyline	5	25.2
	**Mood stabilizers**		**27**	**23.7**
		Carbamazepine	17	51.3
		Lithium	6	27.1
		Sodium valproate	3	16.2
		Lamotrigine	1	5.4
	**Combination of medications**		**20**	**17.5**
		Antipsychotic + Mood Stabilizer	13	45.0
		Antipsychotic + Antipsychotic	7	55.0

### Determinates and impact of mental health treatment on survival outcomes

The findings of Cox regression analysis showed that the presence of comorbid conditions and mental health treatment were significantly associated with hospital readmission, while other variables including sociodemographic factors (age, sex, residence) and clinical parameters( diagnosis and type of medications ), did not show a significant association with the outcome. Patients who did not receive mental health treatment had a 3.44 times higher risk of hospital readmission compared to those who received treatment, with an adjusted hazard ratio (AHR) of 3.44 (95% CI: 2.11–5.62; *P* < 0.001). Moreover, patients with comorbid conditions had a 1.53 risk of hospital readmission compared to those without comorbidities, with an AHR of 1.53 (95% CI: 1.03–2.28; *P* = 0.03) (Table 3).

**Table 3:** Determinants’ of time to hospital readmission among the study participants

					Hospital readmission		Cox Regression Analysis					
							Bivariable analysis			Multivariable analysis		
				Variables	occurred (108)	censored (211)	*P* value	CHR	CI	*P* Value	AHR	CI
Age												
				< 56 years	39	73	0.618	1.10	0.74–1.64			
				≥ 56 years	69	138						
Sex												
				Male	60	110	0.436	1.16	0.79–1.70			
				Female	48	101						
Residence												
				Rural	43	82	0.835	0.96	0.65–1.41			
				Urban	65	129						
Diagnosis												
		Mental health disorders										
		Depression (F32)										
				No	58	110		1				
				Yes	50	101	0.582	0.89	0.61–1.31			
		Anxiety (F41)										
				No	74	156		1			1	
				Yes	34	55	0.18	1.32	0.87–1.98	0.47	1.17	0.76–1.79
		Bipolar disorder (F31)										
				No	88	185		1				
				Yes	20	26	0.30	1.28	0.79–2.08			
		Schizophrenia (F20)										
				No	96	190		1			1	
				Yes	12	21	0.00	2.00	1.23–3.25	0.28	0.68	0.33–1.38
Cardiovascular diseases												
		Hypertension (I10)										
				No	64	136		1			1	
				Yes	44	75	0.23	1.26	0.85–1.85	0.26	1.25	0.84–1.85
			Coronary artery disease (I25.1)									
				No	84	162		1				
				Yes	24	49	0.75	1.07	0.68–1.69			
			Heart failure (I50)									
				No	88	183		1				
				Yes	20	28	0.30	1.28	0.79–2.09			
			Atrial fibrillation (I48)									
				No	98	190		1				
				Yes	10	21	0.62	0.85	0.44–1.63			
			Peripheral artery disease (I73.9)									
				No	99	198	0.37	1.36	0.68–2.69			
				Yes	9	13		1				
			Stroke (I63)									
				No	98	202		1			1	
				Yes	10	9	0.14	1.62	0.84–3.12	0.58	1.20	0.61–2.38
			Valvular heart disease (I34)									
				No	107	205		1				
				Yes	1	6	0.44	0.46	0.06–3.32			
Presence of comorbid conditions												
				No				1			1	
				Yes			0.01	1.61	1.10–2.37	0.03*	1.53	1.03–2.28
Receive mental health treatment												
				No	86	119	0.00	3.31	2.06–5.30	0.00*	3.44	2.11–5.62
				Yes	22	92					1	
Group of psychotropic medications												
	Antipsychotics											
				No	101	183	0.07	2.03	0.94–4.36	0.30	1.51	0.68–3.37
				Yes	7	28		1				
	Antidepressants											
				No	101	186	0.20	1.63	0.76–3.52	0.22	1.61	0.74–3.49
				Yes	7	25		1				
	Mood stabilizers											
				No	96	196	0.31	0.73	0.40–1.33			
				Yes	12	15		1				
	Antipsychotic + Mood Stabilizer											
				No	104	202	0.75	1.17	0.43–3.17			
				Yes	4	9		1				
	Antipsychotic + Antipsychotic											
				No	104	208	0.10	0.44	0.16–1.19	0.12	0.40	0.12–1.27
				Yes	4	3						

*statically significant, CI confidence interval, AHR adjusted hazard ratio, CHR crude hazard ratio

Regarding emergency department visit, findings of Cox regression analysis indicated patients who did not receive mental health treatment had 2.11 times likely to visit the emergency department compared to those who received treatment with an AHR of 2.11 (95% CI: 1.09–4.08; *P* = 0.02). However, no significant association was found between age, sex, residence, diagnosis, type of medications and outcome variable (Table 4).

**Table 4:** Determinants’ of time to emergency department visit among the study participants

				Emergency Department Visit		Cox Regression Analysis					
						Bivariable analysis			Multivariable analysis		
	Variables			Occurred (43)	Censored (276)	*P* value	CHR	CI	*P* value	AHR	CI
Age											
	< 56 years			12	100	0.27	0.68	0.35–1.36	0.25	0.674	0.34–1.32
	≥ 56 years			31	176						
Sex											
	Male			23	147	0.88	1.04	0.57–1.91	0.60	1.172	0.64–2.14
	Female			20	129						
Residence											
	Rural			12	113	0.11	0.58	0.29–1.13	0.06	0.46	0.44–1.10
	Urban			31	163		1			1	
Diagnosis											
	Mental health										
	Depression										
			No	22	146		1				
			Yes	21	130	0.96	1.04	0.55–1.84			
	Anxiety (F41)										
			No	32	198		1				
			Yes	11	78	0.71	1.00	0.50–1.99			
	Schizophrenia (F20)										
			No	41	245		1				
			Yes	2	31	0.30	0.47	0.11–1.95			
	Bipolar disorder (F31)					0.74	1.14	0.50–2.56			
	No			36	237		1				
	Yes			7	39						
	Hypertension (I10)										
			No	24	176		1			1	
			Yes	19	100	0.22	1.44	0.79–2.64	0.43	1.27	0.69–2.34
	Coronary artery disease (I25.1)										
			No	36	210		1				
			Yes	7	66	0.34	0.67	0.30–1.52			
	Heart failure (I50)										
			No	38	233		1				
			Yes	5	43	0.46	0.70	0.27–1.78			
	Atrial fibrillation (I48)										
			No	42	246		1			1	
			Yes	1	30	0.11	0.20	0.02–1.45	0.13	0.21	0.02–1.58
	Peripheral artery disease (I73.9)										
			No	40	257		1				
			Yes	4	18	0.43	1.50	0.53–4.22			
	Stroke (I63)										
			No	42	158		1				
			Yes	2	17	0.32	0.37	0.05–2.70			
	Valvular heart disease (I34)										
			No	42	270		1				
			Yes	1	6						
Presence of comorbid condition											
	No			28	188		1				
	Yes			15	88	0.44	1.28	0.68–2.40			
Receive mental health treatment											
	No			30	175	0.04	1.96	1.01–3.78	0.02*	2.11	1.09–4.08
	Yes			13	101		1				
Group of psychotropic medications											
	Antipsychotics										
		No		38	246	0.94	1.03	0.47–2.63			
		Yes		5	30		1				
	Antidepressants										
		No		35	252	0.05	0.46	0.21–1.01	0.05	0.47	0.21–1.01
		Yes		8	24		1				
	Mood stabilizers										
		No		39	253	0.85	0.90	0.32–2.54			
		Yes		4	23		1				
	Antipsychotic + Mood Stabilizer										
		No		41	265	0.73	0.77	0.18–3.23			
		Yes		2	11		1				
	Antipsychotic + Antipsychotic										
		No		42	270	0.67	0.65	0.08–4.74			
		Yes		1	6		1				

*statically significant, CI confidence interval, AHR adjusted hazard ratio, CHR crude hazard ratio

### Impact of mental health treatment on patient outcomes

Kaplan–Meier survival analysis demonstrated that patients who received mental health treatment had longer median times to both hospital readmission and emergency department (ED) visits compared to those who did not receive such treatment. Specifically, the median time to hospital readmission was 18.5 months in the treatment group versus 14.2 months in the no-treatment group. Similarly, the median time to an ED visit was 20.1 months versus 17.3 months, respectively.

The adjusted Cox regression analysis showed that mental health treatment was significantly associated with a reduced hazard of hospital readmission (adjusted hazard ratio [AHR] = 0.65, 95% CI: 0.43–0.98; *p* = 0.034). One-year and two-year readmission-free survival rates were higher in the treatment group (88% and 79%) compared to the no-treatment group (76% and 68%). For emergency department visits, the hazard was also lower among patients who received mental health treatment (AHR = 0.76, 95% CI: 0.49–1.18); however, this finding did not reach statistical significance (*p* = 0.088). One-year and two-year ED visit-free survival rates were 91% and 83% for the treatment group versus 85% and 77% for the no-treatment group, respectively. (Supplementary Table 1)

The Kaplan-Meier survival curves illustrate the time to hospital readmission among patients who received mental health treatment compared to those who did not. At baseline, both groups began with a cumulative readmission-free survival probability of 1.0. However, as time progresses, the survival curve for patients without mental health treatment (blue line) declined more rapidly than that for patients who received treatment (red line), indicating a shorter time to readmission. In contrast, those who received mental health treatments exhibited a slower decline in survival probability, indicating a reduced readmission rate and longer time to readmission.

**Fig. 1:**
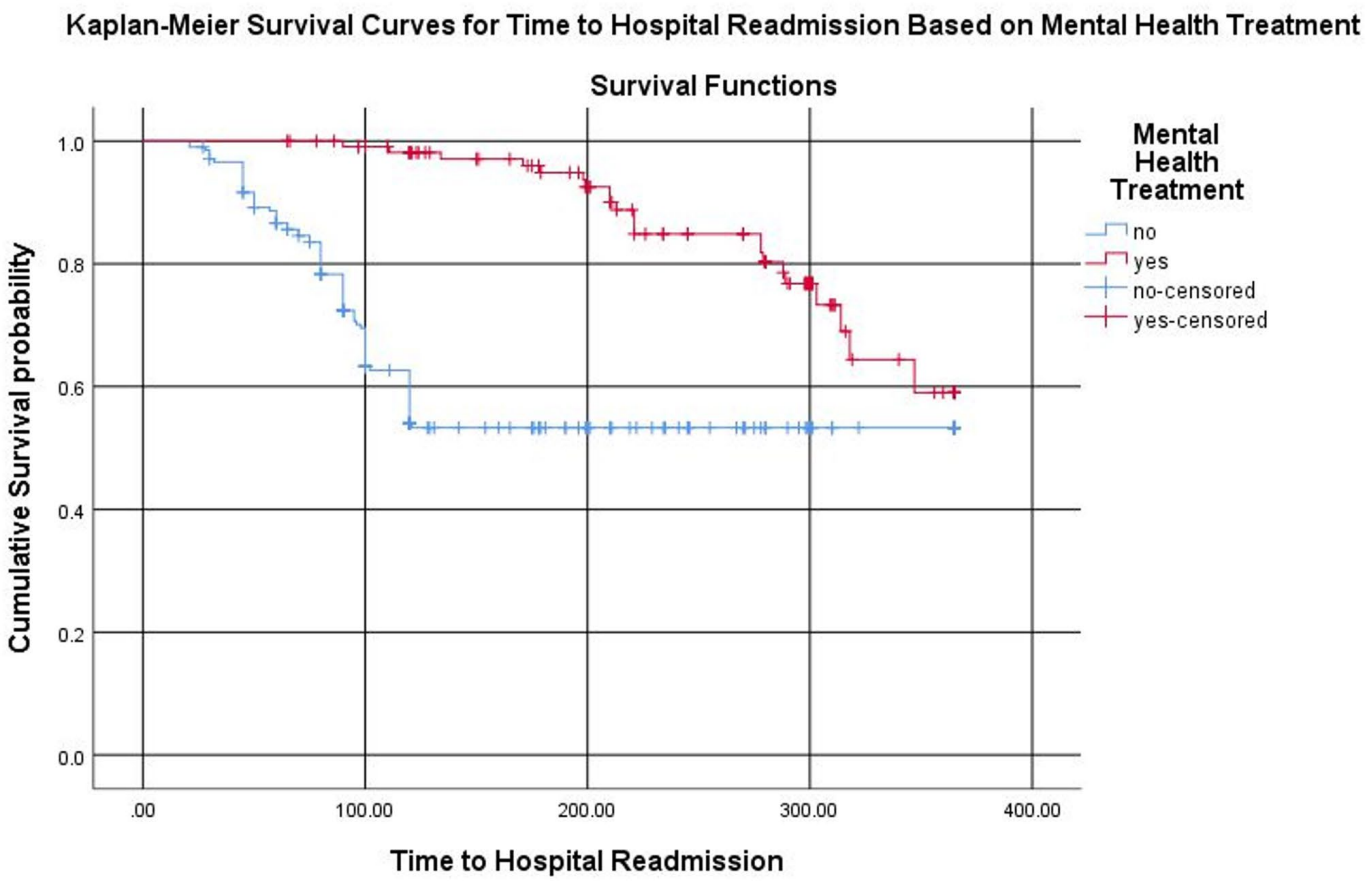
Kaplan-Meier survival curves for time to hospital readmission among patients with and without mental health treatment (log-rank test, *P* = 0.034; HR = 0.65, 95% CI: 0.43–0.98)

Censored patients, represented by “+” symbols on the curves, are those who did not experience a hospital readmission event during the observation period. (Fig. 1; Supplementary Table 1).

**Fig. 2:**
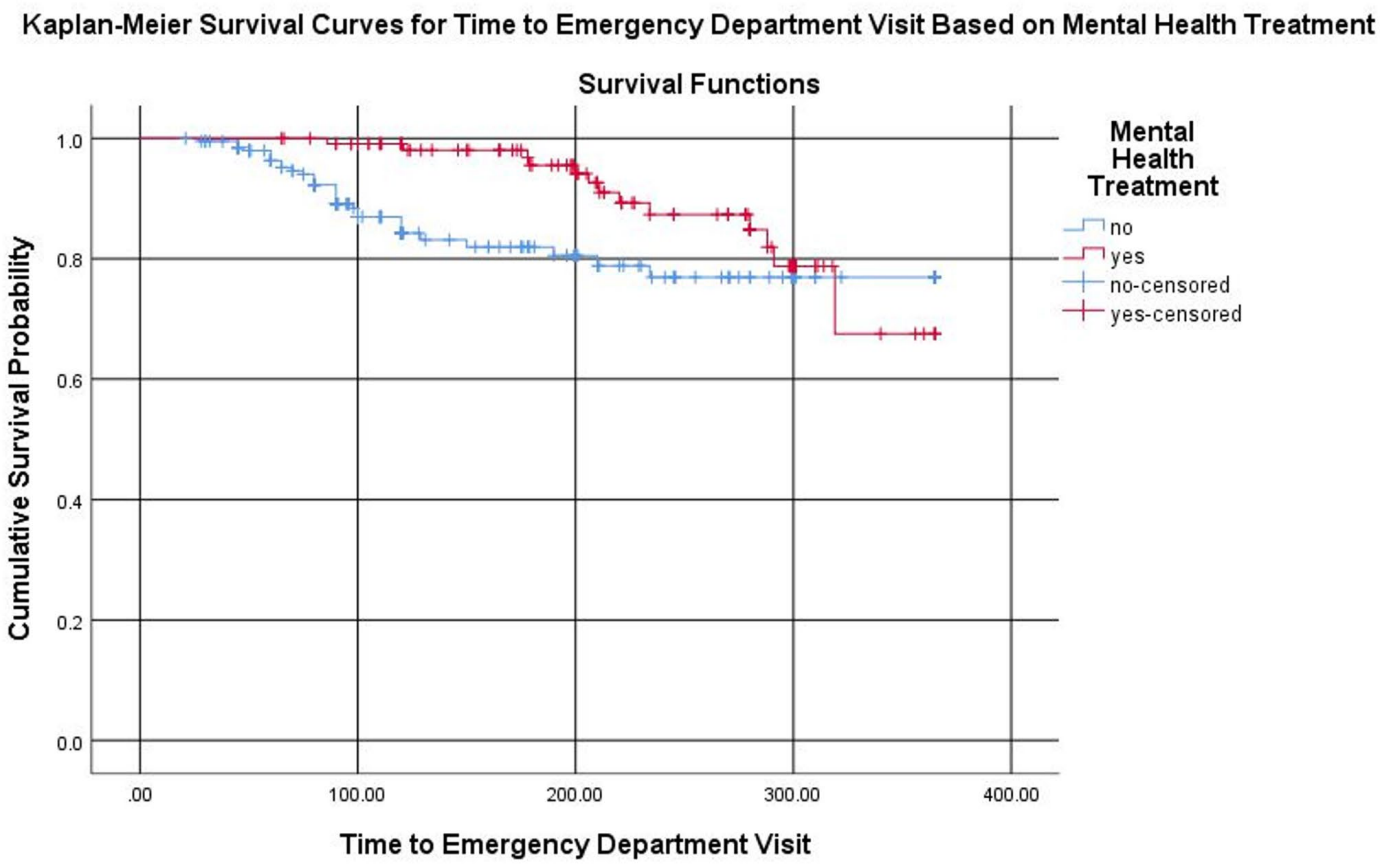
Kalpan-Meier survival curves for time to emergency department visit among patients with and without mental health treatment (log-rank test, *P* = 0.088; HR = 0.76, 95% CI: 0.49–1.18)

A similar trend was observed for emergency department visits, patients receiving mental health treatment (red curve) had a slower decline in survival probability, suggesting longer times to ED visits compared to untreated patients (blue curve). Although this trend favored mental health treatment, the difference between the two groups was not statistically significant. Censored cases, patients who did not experience an ED visit during the follow-up period, are indicated by “+” symbols on the survival curves (Fig. 2; Supplementary Table 1).

## Discussion

Chronic physical conditions, such as cardiovascular disease, are frequently accompanied by psychiatric symptoms or emotional and psychological distress [28, 29]. To our knowledge, this study is the first to assess the impact of mental health treatment and its associated factors in patients with both mental and cardiovascular conditions in Ethiopia.

In this study depression was the most common diagnosis, affecting 47.3% of the patients. This high prevalence aligns with numerous studies that also identify depression as a leading mental health condition among patients with chronic diseases, especially those with cardiovascular comorbidities [30, 31, 32, 33]. Chronic conditions, particularly CVD, are known to significantly increase the risk of developing depression due to various biological, psychological, and social factors that exacerbate both physical and mental health issues. First, chronic diseases like CVD often result in physical limitations and a reduced quality of life, which increase the likelihood of depression. For instance, patients with heart disease frequently suffer from fatigue, pain, and restricted mobility, factors that contribute to feelings of hopelessness and despair. The continuous management of symptoms and medications, along with fear of mortality, places a substantial psychological burden on patients, raising their risk of depression [34]. In our study, depression was particularly prevalent among patients with hypertension (37.3% ) and coronary artery disease (22.9%), conditions that have been strongly associated with depression in previous research [35]. Second, chronic inflammation, commonly seen in cardiovascular diseases, is increasingly recognized as a biological contributor to depression. Elevated levels of pro-inflammatory cytokines in these conditions can disrupt neurotransmitter systems, such as serotonin and dopamine pathways, which are crucial for mood regulation [36]. Moreover, the bidirectional relationship between depression and CVD further complicates the clinical outcomes. Each condition increases the risk of developing the other, and they often co-occur [37]. Depression is associated with poor adherence to treatment, unhealthy lifestyle behaviors like smoking, poor diet, and physical inactivity, all of which worsen chronic illness [38]. In our study, the high number of patients receiving antidepressants (47.7% of whom were prescribed sertraline) shows growing clinical awareness of this issue and the need for integrated care that address both the mental and physical health. Social factors such as isolation, unemployment, and limited social support further contribute to depression among patients with chronic illnesses. In low-resource settings, like Ethiopia, these challenges may be further exacerbated by limited access to mental health services, societal stigma, and a lack of integrated care systems that address both physical and mental health needs [39]. The frequent occurrence of anxiety and bipolar disorder in our population (28.0% and 14.4%, respectively) mirrors findings from other studies that report the high burden of these mental health conditions in patients with physical comorbidities [4]. These disorders are known to worsen physical health outcomes and increase healthcare utilization. Our findings support this, showing that mental health treatment is associated with improved outcomes in terms of hospital readmission and emergency department visits.

Despite the high burden of mental illness, our study found that suboptimal proportion of patients received mental health treatment, similar to findings in previous studies [40]. This shows a significant gap in the provision of mental health care. Although the benefits of mental health interventions are well established, low treatment rates reflect persistent barriers such as inadequate healthcare infrastructure, limited training for healthcare providers, and fragmented care systems in resource limited settings [21]. Research shows that patients with mental health disorders who experience cardiovascular events are more likely to be readmitted to the hospital than those without mental health issues [41]. The interplay between psychiatric and cardiovascular illness often leads to a cycle of deterioration and frequent hospitalizations due to complications in either condition [7, 11]. Patients with both mental and cardiovascular disorders frequently present to emergency departments with acute symptoms, suggesting a lack of comprehensive outpatient care. Such patients are at greater risk of repeated emergency department visits, which strains healthcare systems and increased costs [42].

This study also explored the impact of mental health treatment on readmission rates and emergency department (ED) visits. Our results demonstrates a significant association, Patients who did not receive mental health interventions were three times more likely to be readmitted compared to those who did received treatment. This aligns with prior research indicating that untreated psychiatric symptoms exacerbate physical illness, increase symptom burden, and lead to higher hospitalization rates [43, 44, 45, 46]. Furthermore, 13.5% of the participants had ED visits, and mental health treatment was associated with a twofold reduction in ED visit risk. This reinforces the importance of addressing mental health to improve overall healthcare utilization. Previous studies [46, 47], similarly emphasized that untreated mental illness contributes to higher ED visits due to poor disease management and increased somatic complaints.

The Kaplan-Meier survival analysis in our study further illustrated the positive impact of mental health treatment on survival outcomes. Patients who received mental health treatment had a higher cumulative survival probability, indicating fewer hospital readmissions and ED visits over time. The steeper decline in survival among untreated patients indicates the critical role of mental health care in improving long term outcomes and reducing the frequency of acute care events. Overall, the survival analysis suggests that mental health treatment prolongs survival by reducing the risk of hospital readmission and ED utilization.

In summary, our findings emphasize the crucial role of mental health treatment in reducing hospital readmissions and emergency department visits, which are key markers of healthcare utilization and patient survival. The significant associations found in our study reinforce the need to integrate mental health services into chronic disease management. Our results align with existing literature and underscore the importance of addressing both mental and physical health to improve clinical outcomes. Future research should continue to explore the underlying mechanisms driving these relationships and focus on strategies to enhance access and quality of mental health care for indivuals with chronic diseases.

### Limitation

Although this study is the first of its kind it has limitations. Due to the nature of retrospective data, causal relationships between mental health treatment and survival outcomes cannot be definitively established. Moreover, the study focused exclusively on pharmacological treatments; non-pharmacological interventions, such as psychotherapy, counseling, or community-based mental health services, were not included due to inconsistent or missing documentation in medical records. In addition, we were unable to adjust for certain potentially important confounders, such as medication adherence, access to care, treatment continuity, and socioeconomic status, as these variables were either not recorded or incompletely documented in the source data. This may have introduced residual confounding and affected the precision of the estimated associations. Furthermore, cardiovascular treatment variables were not included in the analysis, as this was beyond the primary aim of the study, which specifically examined the impact of mental health treatment.

Future prospective studies are recommended to collect more comprehensive data, including both pharmacological and non-pharmacological interventions, as well as detailed measures of adherence, healthcare access, and patient-level factors. Doing so would allow for more robust confounder control and enhance the understanding of how different aspects of mental health treatment influence cardiovascular outcomes and healthcare utilization.

## Conclusion

This study indicates the critical role of mental health treatment in improving outcomes for patients with chronic diseases, particularly those with cardiovascular conditions. Untreated mental health issues were significantly associated with higher risks of hospital readmissions and emergency department visits. Depression was the most common mental health disorder, emphasizing the need for integrated care. Moreover mental health interventions significantly reduced acute care events and improved survival outcomes. These findings reinforce the necessity of integrating mental health services into routine care to enhance patient outcomes, reduce healthcare utilization, and address barriers to access, particularly in resource-limited settings.

## Electronic supplementary material

Below is the link to the electronic supplementary material.

Supplementary Material 1

## Data Availability

All the necessary data is included in the manuscript. On request, the corresponding author can give any additional details.

## Abbreviations

CVD: Cardiovascular diseases
ED: Emergency department
