# Supplementary material for: Mental health treatment and its impact on survival outcomes in patients with comorbid mental health and cardiovascular diseases: a retrospective cohort study
Source: BMC Psychiatry. 2025 Jul 1;25:609. doi: 10.1186/s12888-025-07035-4 (PMC12210634; doi:10.1186/s12888-025-07035-4)
Supplement: Supplementary file 1 — Supplementary Material 1 [file 12888_2025_7035_MOESM1_ESM.docx]

Supplementary Table 1. Cox Regression and Kaplan–Meier Survival Estimates for Hospital Readmission and Emergency Department Visit

| Outcome | Comparison Group | Hazard Ratio (95% CI) | Log-rank P-value | Median Survival Time (months) | 1-Year Survival (%) | 2-Year Survival (%) |
| --- | --- | --- | --- | --- | --- | --- |
| Hospital Readmission | Mental health treatment vs. no treatment | 0.65 (0.43–0.98) | 0.034 | 18.5 vs. 14.2 | 88% vs. 76% | 79% vs. 68% |
| Emergency Department Visit | Mental health treatment vs. no treatment | 0.76 (0.49–1.18) | 0.088 | 20.1 vs. 17.3 | 91% vs. 85% | 83% vs. 77% |

Note: Hazard ratios are from Cox proportional hazards regression models. Median survival times represent the estimated time (in months) at which 50% of participants experienced the event.
